# Complete genome sequences of the prosthecate dimorphic bacteria *Brevundimonas intermedia* CB63^T^ and *Brevundimonas staleyi* FWC43^T^

**DOI:** 10.1128/mra.01050-24

**Published:** 2024-11-20

**Authors:** Joel Hallgren, Fredrik Daneby, Kristina Jonas

**Affiliations:** 1 Department of Molecular Biosciences, The Wenner-Gren Institute, Science for Life Laboratory (SciLifeLab), Stockholm University, Stockholm, Sweden; SUNY College of Environmental Science and Forestry, Syracuse, New York, USA

**Keywords:** *Brevundimonas*, dimorphic prosthecate bacteria, freshwater, plasmid

## Abstract

Here, we report the complete genome sequences of the dimorphic prosthecate bacterial species type strains *Brevundimonas intermedia* CB63^T^ and *Brevundimonas staleyi* FWC43^T^, isolated from pond water and wastewater, respectively. *B. intermedia* CB63^T^ contains a chromosome of 3.60 Mb, and *B. staleyi* FWC43^T^ contains a chromosome of 3.97 Mb and a 157-kb plasmid.

## ANNOUNCEMENT

Dimorphic prosthecate bacteria (DPB) are ubiquitous in water and soil (1) and are employed as models for studying bacterial cell development (2). *Brevundimonas intermedia* CB63^T^ is an unpigmented vibrioid DPB isolated by Jeanne Poindexter from pond water in California (USA) in 1962 (3). *B. staleyi* FWC43^T^ is a yellow-pigmented bacteroid DPB isolated from activated sludge of a secondary wastewater treatment facility in Calgary (Canada) (4). We have sequenced these type strains to aid taxonomy and comparative genomics.

Lyophilized cultures of *B. intermedia* CCUG 45022^T^ (= CB63^T^) and *B. staleyi* CCUG 57884^T^ (= FWC43^T^) were obtained from the Culture Collection University of Gothenburg (CCUG), rehydrated in 100 µL broth and transferred onto agar plates; peptone yeast extract (PYE) medium (2 g/l Bacto peptone, 1 g/l Bacto yeast extract, 1 mM MgSO_4_, 0.5 mM CaCl_2_; solidified with 15 g/l Bacto agar) was used for *B. intermedia* and modified PYE (PYEM) (4) for *B. staleyi*. After 3 days of incubation (30°C) plus an additional day (23°C) for *B. staleyi*, 10 mL broth cultures were inoculated with single colonies and then preserved in 10% DMSO at −80°C after 1 day (30°C, 200 rpm shaking). Frozen stocks were streaked onto PYE and incubated for 5 days (23°C) before inoculating single colonies into 25 mL PYE broth. Cells were harvested from ~5 mL of exponentially growing cultures (after 2 days, 23°C, 200 rpm), and DNA was extracted using Genomic tips 20/G (Qiagen, Germany) following the manufacturer’s instructions for Gram-negative bacteria, which includes an RNase A treatment step (5). DNA was transferred with glass hooks to low TE buffer pH 8.0 (1 mM Tris-HCl and 0.1 mM EDTA) following isopropanol precipitation and solubilized with gentle agitation at 23°C overnight followed by 50°C for ~12 hours and consecutive addition of low TE buffer.

Genomic DNA was sequenced at the National Genomics Infrastructure in Uppsala (Sweden) using the Revio system (102-090-600) (Pacific Biosciences, USA). For this, 5 µg DNA was sheared into 11–16-kb fragments using Megaruptor 3 (Diagenode, USA), and fragments below 10 kb were removed using a PippinHT (Sage Science, USA). The sequencing library was prepared using the SMRTbell prep kit 3.0 (Pacific Biosciences, USA) and sequenced on the Revio single-molecule real-time cell platform (25M) (Pacific Biosciences, USA) using 0.277 µg for primer annealing and a Revio polymerase kit (102-817-600) (Pacific Biosciences, USA) for polymerization. For *B. intermedia* CCUG 45022^T^ and *B. staleyi* CCUG 57884^T^, respectively, 217,797 reads yielding 3,306,752,143 bp and 210,490 reads yielding 3,241,089,915 bp were obtained. To reduce the amount of data for assembly to <100×, reads were split into nine equally sized data sets (24,200 and 23,388 reads, respectively), and one such data set was used for assembly with Hifiasm version 0.19.6 (6, 7) (default parameters). Another data set was then assembled separately for verification. Sequences were annotated using the NCBI Prokaryotic Genome Annotation Pipeline (8).

For *B. intermedia* CCUG 45022^T^, genome assembly resulted in a single circular chromosome contig (3,604,506 bp; 83× mean coverage depth; 67% G + C; 3,452 predicted protein-coding sequences [CDSs]; 50 tRNAs; and 2 rRNA suites [5S, 16S, and 23S]). For *B. staleyi* CCUG 57884^T^, it resulted in a circular chromosome contig (3,970,337 bp; 75× mean coverage depth; 68% G + C; 3,919 CDSs; 46 tRNAs; and 2 rRNA suites) and a circular plasmid contig (156,797 bp; 37× mean coverage depth; 68% G + C; 157 CDSs; no tRNAs; and no rRNAs).

## Data Availability

Genome projects, including sequencing reads, are available under BioProject accession numbers PRJNA1155294 and PRJNA1155342. The *B. intermedia* CCUG 45022^T^ chromosome sequence can be found under the GenBank accession number CP169067, and the *B. staleyi* CCUG 57884^T^ chromosome and plasmid sequences under CP169082 and CP169083, respectively.
